# HIV-associated Kaposi’s sarcoma in Maputo, Mozambique: outcomes in a specialized treatment center, 2010–2015

**DOI:** 10.1186/s13027-018-0177-6

**Published:** 2018-01-19

**Authors:** Vini Fardhdiani, Lucas Molfino, Ana Gabriela Zamudio, Rolanda Manuel, Gilda Luciano, Iza Ciglenecki, Barbara Rusch, Laurence Toutous Trellu, Matthew E Coldiron

**Affiliations:** 1 Médecins Sans Frontières, Maputo, Mozambique; 2 Ministry of Health, Maputo, Mozambique; 3 Médecins Sans Frontières, Geneva, Switzerland; 4 Hôpitaux Universitaires de Genève, Geneva, Switzerland; 5 Epicentre, 8 rue Saint-Sabin, 75011 Paris, France

**Keywords:** Kaposi sarcoma, Acquired immunodeficiency syndrome, AIDS-related opportunistic infections, Doxorubicin, Mozambique

## Abstract

**Background:**

Kaposi’s sarcoma (KS) is a common HIV-associated malignancy associated with disability, pain and poor outcomes. The cornerstone of its treatment is antiretroviral therapy, but advanced disease necessitates the addition of chemotherapy. In high-income settings, this often consists of liposomal anthracyclines, but in Mozambique, the first line includes conventional doxorubicin, bleomycin and vincristine, which is poorly-tolerated. Médecins Sans Frontières supports the Ministry of Health (MOH) in a specialized HIV and KS treatment center at the Centro de Referencia de Alto Maé in Maputo.

**Methods:**

We performed a retrospective analysis of data collected on patients enrolled at the CRAM between 2010 and 2015, extracting routinely-collected clinical information from patient care databases. KS treatment followed national guidelines, and KS staging followed AIDS Clinical Trials Group and MOH criteria. Baseline description of the cohort and patient outcomes was performed. Risk factors for negative outcomes (death or loss to follow-up) were explored using Cox regression.

**Results:**

Between 2010 and 2015, 1573 patients were enrolled, and 1210 began chemotherapy. A majority were young adult males. At enrollment, CD4 was < 200 cells/μl in 45% of patients. Among patients receiving chemotherapy, 78% received combination doxorubicin-bleomycin-vincristine. Among patients receiving chemotherapy, 43% were lost to follow-up and 8% were known to have died. In multivariate regression, the only risk factors identified with poor outcomes were CD4 < 100 cells/μl at enrollment (Risk ratio 1.5, 95%CI 1.1–2.1, *p* = 0.02 and having S1 disease (RR 1.7, 95%CI 1.2–2.3, *p* = 0.001).

**Discussion:**

We describe a large cohort of patients receiving care for HIV-associated KS in a specialized clinic in an urban setting. Outcomes were nonetheless unsatisfactory. Efforts should be made to decrease late referrals and entry into care and to increase access to more effective and better-tolerated treatments like liposomal doxorubicin.

## Introduction

Kaposi’s sarcoma (KS) is the most frequent cancer among persons living with HIV/AIDS in high-resource settings [1] and is a major cause of mortality in sub-Saharan Africa [2]. In Mozambique, it is the most frequent cancer documented in cancer registers in the major cities of Maputo and Beira [3], with an estimated age-standardized incidence of 23 cases per 100,000 persons per year and an estimated 3215 deaths due to KS in Mozambique in 2012 alone [4].

KS is an AIDS-defining opportunistic infection and is associated with human herpes virus 8 (HHV8) infection. Advanced stage KS may cause considerable morbidity due to edema and skin and soft tissue infection, ulceration and necrosis; visceral lesions can cause acute, life-threatening bleeding [5, 6]. And while its classic presentation in non-immunosuppressed individuals is often indolent, current experience in sub-Saharan Africa suggests that KS can be aggressive and lead to poor outcomes [7]. The fact that KS lesions are often visible and quite painful often leads to both physical and psychological disability [8], and indeed the outcomes of some clinical trials have been based on quality of life [9].

The AIDS Clinical Trials Group (ACTG) staging system for AIDS-KS classifies patients based on three variables: tumour extent (T), immune system status (I), and evidence for HIV-associated systemic illness (S). Each variable is classified as good risk (0) or poor risk (1) [10]. These prognostic factors have been prospectively and independently validated [11].

The treatment of HIV-associated KS is based on reconstituting the immune system with antiretroviral therapy (ART), which has been shown to significantly reduce the burden of KS disease by itself [12, 13]. Nonetheless, when disease is advanced, it is often necessary to add cytotoxic chemotherapy to control KS disease more quickly [14]. This is often the case in sub-Saharan Africa, where patients present with advanced disease. Several agents are effective against HIV-associated KS, including doxorubicin, vincristine, bleomycin and etoposide. In high-income countries, standard first-line chemotherapy consists of liposomal anthracyclines or taxanes [6, 15]. Pegylated liposomal formulations of doxorubicin (PLD) are associated with more favourable outcomes and safety profiles than conventional forms [16–19], and allow for monotherapy. However, in low- and middle-income countries, a combination of bleomycin and vincristine, with or without conventional doxorubicin (ABV) are more often the standard of care due to high cost and unavailability of liposomal formulations. These combinations are more poorly-tolerated and associated with lower quality of life [9].

Access to cancer treatments, including KS, remains limited in many sub-Saharan African settings [20]. General health systems barriers include lack of medications, lack of clear guidelines, clinical staff with little oncological training, a dearth of diagnostic and monitoring tools and poor infrastructure necessary to handle cytotoxic medications [21–23]. Beyond these general concerns, KS presents a few specific challenges in low-resource settings. Decision-making about when to start and stop treatment is not easily-standardized because of the beneficial effects of ART alone, and also because of side effects associated with ABV, the poor availability of second-line cytotoxic treatments, and because of the KS immune reconstitution inflammatory syndrome [24, 25].

To address the burden of HIV-associated KS, Médecins Sans Frontières (MSF), in collaboration with the Ministry of Health of Mozambique (MOH), established a KS treatment unit in Maputo in 2010. In this report, we describe a large cohort of HIV-infected KS patients and their clinical outcomes. We further explore risk factors for negative outcomes such as death and loss to follow-up.

## Methods

### Study setting

The Centro de Referência de Alto-Maé (CRAM) was established in 2009 as a collaboration between the MOH and MSF in Chamanculo Health District, in Mozambique’s capital, Maputo. The goal was to bridge the gap between primary health care and referral hospitals by ensuring access to specialized care for HIV-infected patients with complications (such as advanced disease, treatment failure and KS). CRAM provides a comprehensive package of medical, laboratory, and psychological care, including second- and third-line ART for adults and children, chemotherapy for HIV-associated KS, diagnosis and treatment of OIs, and management of side effects of ART.

### KS care and treatment

CRAM officially serves an urban area with a population of approximately 350,000 persons, but given that it is one of only three KS treatment centers in Maputo, it treats many patients from outside its official catchment area. Since 2013, the pharmaceutical department of Geneva University Hospitals has supported the effective and safe management of cytotoxic drugs at CRAM, including training of clinical and paraclinical staff, infrastructure development and waste disposal.

Patients with suspected HIV-associated KS undergo a thorough clinical history and examination and are staged according to Mozambican guidelines and ACTG criteria. Historically, most dermatologic KS has been diagnosed clinically due to limited availability of histopathology. All cases where the clinical diagnosis is uncertain, and all cases with suspected visceral involvement, are referred to the tertiary-level hospital for biopsy and other invasive procedures such as bronchoscopy and endoscopy.

Care in the CRAM follows current treatment recommendations in Mozambique, which call for ART for T0 disease, and if no response after 6 months, to begin systemic chemotherapy with bleomycin and vincristine every 3–4 weeks [26]. In the case of T1 disease, in addition to ART, therapy with doxorubicin, vincristine and bleomycin (ABV) is started immediately, with cycles every 3–4 weeks. For T1 patients not on ART, a delay of 2–3 weeks is recommended between initiation of ART and chemotherapy. Serum haemoglobin levels and the absolute neutrophil count are routinely monitored prior to each dose of chemotherapy. Chemotherapy is continued until remission of all cutaneous and mucosal lesions, and relief of obstructions and symptoms, including pain, improvement in function, or until the cumulative dose of 550 mg/m^2^ of doxorubicin or 440 UI of bleomycin is reached. Patients with disease progression despite the ABV therapy are referred to the dermatology department at the tertiary hospital for second-line chemotherapy.

CRAM also provides primary HIV care (ARTs, education, adherence counselling) as well as psychosocial care for its patients. KS patients also have dedicated services for wound care and analgesia. A system for tracing patients lost to follow-up through phone calls and home visits is also in place.

### Study design and outcome definitions

We performed a retrospective cohort study on patients co-infected with HIV/KS, and beginning treatment with ART and/or chemotherapy from January 1, 2010 to December 31, 2015 at CRAM. Data were extracted from the routine clinical databases maintained for follow-up of patient care. Outcomes and treatment responses were judged every 3–6 cycles after initiation of KS chemotherapy per national recommendations, and therefore not routinely captured for patients who do not receive chemotherapy. Complete response (CR) is defined as resolution of any detectable disease for at least 4 weeks, including KS-associated oedema or effusion. Partial response (PR) is defined as the absence of new cutaneous, oral or visceral lesions, or the absence of worsening of tumour-associated oedema and effusions, for greater than 4 weeks. In addition, at least one of the following criteria is necessary: (1) 50% decrease in the number of previously existing skin lesions; (2) at least 50% of flattening of all previously raised lesions; (3) 50% decrease in the sum of the products of the largest perpendicular diameters of indicator lesions selected at enrolment; or (4) the patient meets criteria for CR except that residual tumour-associated oedema or effusion is present. Progressive disease is defined as new or progressive visceral lesions, worsening of tumor-related edema for at least one week, a 25% increase in the number of cutaneous lesions. Patients with no contact for a period of 6 months, after at least two attempts at tracing, were considered lost to follow-up.

### Data analysis

Baseline variables and clinical outcomes were described using frequencies and percentages for categorical variables, and means or medians and measures of dispersion for continuous variables. For patients who did not receive chemotherapy, follow-up time began at the date of first visit. For patients who did receive chemotherapy, follow-up time began at the date of the first dose of chemotherapy. Follow-up time ended at the date of death, transfer, or program discharge. For patients lost to follow-up, the date of their last visit was considered their date of study exit. For patients continuing in care, the database was closed on December 31, 2015.

For patients receiving any chemotherapy, survival time until death or loss-to follow-up was described using a Kaplan-Meier survival function.

Cox regression was used to explore factors associated with death and loss to follow up, but patients who were eventually transferred to other centers were excluded from this analysis, since some were transferred for escalation of care while others were transferred because of personal preference. Covariates were included in a multivariate model if *p* < 0.2 in univariate analysis. We combined death and lost to follow-up (LTFU) in the models to more conservatively estimate associations between covariates of interest and the poor outcomes. Two-sided *p*-values < 0.05 were considered significant. All statistical analyses were performed using Stata version 14.1 (College Station, TX, USA).

### Ethics statement

This research fulfilled the exemption criteria set by the Médecins Sans Frontières Ethics Review Board for a posteriori analyses of routinely collected clinical data and thus did not require MSF ERB review. It was conducted with permission from Micaela Serafini, Medical Director, Médecins Sans Frontières – Operational Center Geneva.

## Results

### Description of the cohort

**Fig. 1:**
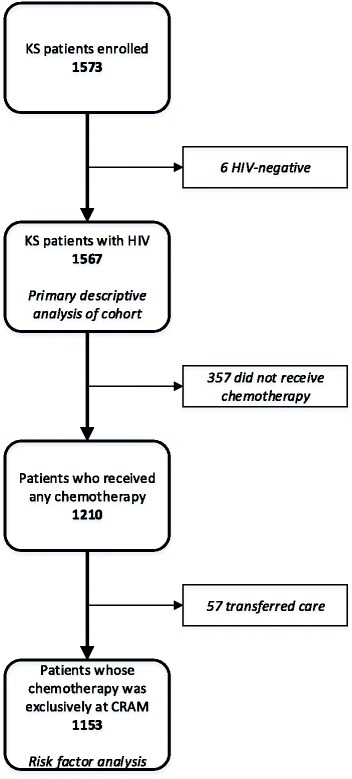
Patients included in analysis

A total of 1573 patients (including 6 HIV-negative patients) were enrolled between 2010 and 2015 (Fig. 1). The 1567 HIV-positive patients form the core clinical cohort which is described. A majority were young adult males (Table 1).

**Table 1:** Baseline clinical description of the study population

Characteristic	n	%
Sex		
Male	1002	64
Female	565	36
Age in years (*N* = 1564)		
< 15	19	1
15–29	405	26
30–44	851	54
≥ 45	289	19
BMI (*N* = 1490)		
< 18.5	177	12
≥ 18.5	1313	88
History of ART (*N* = 1461)		
ART prior to referral	1069	73
No ART prior to referral	392	27
CD4, cells/μl (*N* = 732)		
< 100	181	25
100–199	145	20
200–349	199	27
≥ 350	207	28
Kaposi Sarcoma Stage (*N* = 1474)		
T0S0	192	13
T0S1	54	4
T1S0	913	62
T1S1	309	21

1069 (68%) patients had already started ART prior to program enrolment, but among those patients, 512 (50%) had been on ARV for less than 1 month, and 714 (67%) for less than 6 months. CD4 counts were available for 47% of patients at time of enrolment, of whom 55% had CD4 > 200 cells/μl (Table 1). HIV viral load testing was introduced in 2014, and among eligible patients (those on ARV for > 6 months) with results, only 88/245 (36%) had an undetectable viral load at the time of presentation.

A total of 1210 patients initiated any type of chemotherapy during the study period. Triple therapy with doxorubicin, bleomycin and vincristine was the most common combination of cytotoxic drugs given to patients receiving chemotherapy (Table 2).

**Table 2:** History of Kaposi sarcoma treatment

Initial KS treatment regimen	N	%
Cytotoxic chemotherapy	1210	77
*Doxorubicin-bleomycin-vincristine*	*940*	*78*
*Bleomycin-vincristine*	*174*	*14*
Other chemotherapy regimens*	*96*	*8*
Antiretroviral therapy alone	357	23

*Includes doxorubicin-bleomycin, doxorubicin-vincristine, and ABV-methotrexate

### Response to treatment

**Fig. 2:**
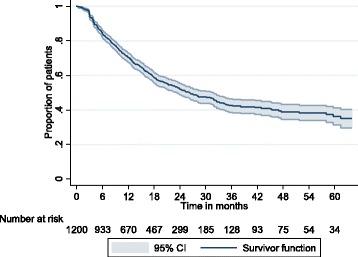
Time until death or loss to follow-up among patients receiving chemotherapy

Among patients receiving chemotherapy, the median length of follow-up time was 14.1 months (IQR 6.5–23.9). A total of 122 (8%) patients were known to have died, but 678 (43%) were lost to follow-up (Table 3, Fig. 2). The proportion of patients lost to follow-up was higher among patients not receiving chemotherapy than among those who did.

**Table 3:** Vital status at time of exit from care

	ARV alone (*N* = 357)		Chemotherapy (*N* = 1210)		Cohort (*N* = 1567)	
Outcome	n	%	n	%	N	%
Died	33	9	89	7	122	8
Known alive	85	24	682	56	767	49
Lost to follow up	239	67	439	36	678	43

**Fig. 3:**
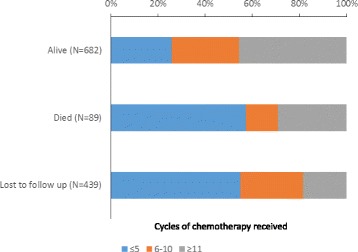
Cycles of chemotherapy received by patient vital status

Among patients receiving chemotherapy known to be alive when they exited care, 312/682 (46%) had received ≥11 cycles. On the other hand, 57% of patients who died and 55% of patients who were lost to follow up did so after receiving ≤5 cycles of chemotherapy (Fig. 3).

Information on disease progression among patients receiving chemotherapy is incomplete, but is available for 517 patients, of whom 62 (12%) achieved complete remission and 448 (87%) ever achieved partial remission. A total of 5 patients had stable disease after treatment and 2 had progressive disease. Among patients achieving complete or partial remission, the median number of cycles of chemotherapy received was 12 (IQR 8–17, range 1–27).

Having a CD4 < 100 cells/μl and having S1 disease at presentation were significantly associated with poor outcomes (loss to follow-up or death). No other risk factors were associated with poor outcomes in univariate or multivariate analysis (Table 4).

**Table 4:** Factors associated with death or loss to follow-up (*N* = 1153)

		Univariate Analysis			Multivariate Analysis		
Characteristic	n (%)	HR	95% CI	*p*	HR	95% CI	*p*
Sex							
Female	416 (36)	ref			ref		
Male	737 (64)	1.1	0.9–1.4	0.16	1.1	0.8–1.5	0.6
Age in years							
< 35	599 (52)	ref					
≥ 35	554 (48)	0.9	0.8–1.1	0.35			
Baseline CD4 (cell/mm^3^) (*N* = 583)							
≥ 100	451 (77)	ref			ref		
< 100	132 (23)	1.5	1.1–2.1	0.02	1.5	1.1–2.1	0.02
Tumour extension (*N* = 1120)							
T0	169 (15)	ref					
T1	951 (85)	1.0	0.8–1.3	0.94			
Systemic disease (n = 1120)							
S0	881 (79)	ref			ref		
S1	239 (21)	1.7	1.4–2.0	< 0.001	1.7	1.2–2.3	0.001
ART at baseline							
No	316 (27)	ref					
Yes	837 (73)	1.1	0.9–1.3	0.5			

*HR: hazard ratio

## Discussion

We describe one of the largest cohorts of KS patients in sub-Saharan Africa, treated in an urban secondary-level health facility. This specialty centre has a comprehensive package for the treatment of KS, and is based on collaboration between MOH and MSF, with technical support from the Geneva University Hospital. Nonetheless, despite all of these advantages, our results show many of the challenges inherent with the treatment of KS in Africa today, and suggest a clear agenda for improving quality of care for patients with HIV-associated KS moving forward.

The typical KS patient was a relatively young male who presented with relatively advanced immunosuppression (half had CD4 < 200 at enrolment, and most had started ART in the previous 6 months) and significant burden of KS disease (83% with T1 disease, 25% with S1 disease). These patients are broadly similar to many other cohorts described, in both rural and urban areas, and in primary care and referral-level centers in southern Africa [7, 27, 28] It is also important to point out that only half of our patients had CD4 available at baseline, likely because most presented with Stage IV HIV disease, which did not warrant immediate CD4 measurement according to guidelines during most of the follow-up period. The actual proportion of patients with CD4 < 200 at enrollment was likely to be even higher. In any case, these “late presentations” mean that innovation in screening and early referral both at primary health facilities and also in the community is desperately needed.

Patients arriving with advanced KS disease were at high risk of attrition and had an overall poor prognosis. It is likely in this setting, where patient tracing was incomplete, that many of those patients lost to follow-up represent unrecorded deaths [7]. It is important to note that the contact tracing was performed under routine program conditions, and is not necessarily analogous to the rigorous contact tracing systems that one might expect to find in a clinical trial. Nonetheless, rates of loss to follow-up as high as 40% have been described in other cohorts in sub-Saharan Africa [29], but in other settings, retention in care at one year approaches 80% [28].

Data on side effects and tolerability are not routinely collected in the CRAM, but our subjective experience is that standard ABV is poorly-tolerated by many patients with advanced disease and immunosuppression. In the immediate term, vomiting and the myelosuppressive effects of doxorubicin are frequent; bacterial superinfection and sepsis can delay chemotherapy delivery. In the intermediate term, the development of neuropathies related to vincristine can exacerbate already-severe pain related to the massive lymphedema seen in many patients with advanced disease. In the end, whether poor outcomes are caused by KS, by advanced HIV (or opportunistic infections), or by a poorly-tolerated treatment, we have clearly described a vicious cycle that is difficult to overcome, even in an “ideal” specialized setting.

Data on disease progression was incomplete, so it is difficult to draw definitive conclusions about the effectiveness of treatment. Nonetheless, we note that a median of 12 cycles of chemotherapy to reach remission. This may be partly due to a lack of standardization of disease progression measures in daily clinical practice, but it also likely represents overtreatment. As soon functional capacity has returned, and the patient has appropriate social support, ART alone could be tested, even among patients who begin with advanced disease, as it has been associated with reasonable improvement in quality of life [30]. Treatment response needs to be judged more quickly, and with the understanding that complete remission will be very rare among patients presenting with advanced disease and particularly those with lymphedema. The illogical paradigm of “treat until toxic cumulative doses are reached” should be re-thought. Once pain and edema are brought under control, chemotherapy can be stopped, or at least used less frequently, while full immune reconstitution with ART takes place.

One of the easiest potential solutions to this cycle of problems would be to use simpler chemotherapies such as oral etoposide or lenalidomide for early-stage disease, but lenalidomide has been poorly studied in similar contexts [31], and two recent multicentric trials that included etoposide at African sites have been stopped early because of poor performance of etoposide [32, 33]. Another solution would be to increase access to “gold standard” chemotherapies that are available in the global north, such as PLD or taxanes. PLD has a favourable side effect profile, and its potential for monotherapy make it ideal for settings similar to the one we describe, but its high cost means that it is currently not realistic for widespread use in sub-Saharan Africa. A pilot program using PLD is currently underway in the CRAM; if successful, it could be used to leverage a market and reduce prices. At a health-systems level, this kind of innovation creates opportunities to develop specialized outpatient oncological platforms.

The use of routinely-collected program data reflects realities in the field, and is one of the strengths of our analysis. To our knowledge, cohorts of similar size and length of follow-up have not been reported. Nonetheless, our findings come with limitations related to the nature of the data collection process, which has been fragmented over the years, and which is often seen as being of secondary importance in a busy, real-world program setting. The high proportion of patients lost to follow-up also makes some outcome data difficult to interpret. Future research, including using qualitative methods, is needed for a better understanding of the reasons for loss to follow-up. The results of this single-center, retrospective study may not be generalizable to other settings in sub-Saharan Africa. As one of only three referral centers where chemotherapy is given in Maputo, CRAM likely receives a disproportionate share of severe cases, and outcomes for patients with less-advanced disease, who may be treated in the community with ARV alone, may be different.

## Conclusions

We describe a large cohort of patients with HIV-associated KS in a secondary health facility in a low-resource urban setting. Despite structural advantages of the program, these patients have had relatively poor outcomes with high loss to follow-up. Urgent measures are required to address this, including increasing ART coverage and improving early access to ART, working with clinicians and community members to decrease time to recognition of KS and referral, and changing old treatment protocols to include more effective and better-tolerated chemotherapy such as PLD. This last point will necessarily involve increasing access, lowering price, and obtaining regulatory approvals of newly-appearing generic PLD products. Given the high burden of disease, this should be a main priority of policymakers in the most-affected countries.

## Abbreviations

ABV: Combination therapy with doxorubicin, bleomycin and vincristine
ACTG: AIDS Clinical Trial Group
AIDS: Acquired Immunodeficiency Syndrome
ART: Antiretroviral therapy
CRAM: Centro de Referência de Alto-Maé
HHV8: Human herpesvirus 8
HIV: Human immunodeficiency virus
KS: Kaposi’s sarcoma
LTFU: Loss to follow-up
MOH: Ministry of Health
MSF: Médecins Sans Frontières
PLD: Pegylated liposomal doxorubicin
